# MHCII expression on gut macrophages supports T cell homeostasis and is regulated by microbiota and ontogeny

**DOI:** 10.1038/s41598-023-28554-8

**Published:** 2023-01-27

**Authors:** Joël Guillaume, Andrea Leufgen, Fabian T. Hager, Oliver Pabst, Vuk Cerovic

**Affiliations:** 1 Institute of Molecular Medicine, RWTH Aachen University, Pauwelsstraße 30, 52074 Aachen, Germany; 2 Present Address: Center for Translational Immunology, UMC Utrecht, Utrecht, The Netherlands

**Keywords:** Antigen-presenting cells, MHC class II, Mucosal immunology, Phagocytes

## Abstract

Macrophages are traditionally considered antigen-presenting cells. However, their ability to present antigen and the factors regulating macrophage MHCII expression are poorly understood. Here, we demonstrate that MHCII expression on murine intestinal macrophages is differentially controlled by their residence in the small intestine (SI) or the colon, their ontogeny and the gut microbiota. Monocyte-derived macrophages are uniformly MHCII^hi^, independently of the tissue of residence, microbial status or the age of the mouse, suggesting a common monocyte differentiation pathway. In contrast, MHCII expression on long-lived, prenatally-derived Tim4^+^ macrophages is low after birth but significantly increases at weaning in both SI and colon. Furthermore, MHCII expression on colonic Tim4^+^, but not monocyte-derived macrophages, is dependent on recognition of microbial stimuli, as MHCII expression is significantly downregulated in germ-free, antibiotic-treated and MyD88 deficient mice. To address the function of MHCII presentation by intestinal macrophages we established two models of macrophage-specific MHCII deficiency. We observed a significant reduction in the overall frequency and number of tissue-resident, but not newly arrived, SI CD4^+^ T cells in the absence of macrophage-expressed MHCII. Our data suggest that macrophage MHCII provides signals regulating gut CD4^+^ T cell maintenance with different requirements in the SI and colon.

## Introduction

Macrophages express major histocompatibility class (MHC)II and are traditionally considered antigen-presenting cells, although the extent and level of macrophage MHCII expression varies in different tissues^1^. In particular, intestinal macrophages express high levels of surface MHCII which had, in the past, contributed to their confusion with dendritic cells (DCs)^2–4^. Nevertheless, only intestinal DCs, but not macrophages, migrate in intestinal lymph^4,5^ and are essential for priming of naïve T cells in the draining mesenteric lymph node (MLN)^6^. Sedentary macrophages in the gut lamina propria (LP) are thus unlikely to play a role in priming of naïve T cells. However, it is currently less clear whether MHCII antigen presentation by macrophages can affect local T cell responses, proliferation or survival.

Multiple studies have demonstrated that intestinal macrophages can support the maintenance and survival of local effector CD4^+^ T cells by cytokine secretion^7–9^. In the steady state, intestinal macrophages can also aid the development and function of intestinal regulatory T cells (Tregs) by IL-10 secretion^10,11^ or TGFβ activation^12^. Thus, it has been proposed that interaction of CD4^+^ T cells with tissue macrophages may act as a second checkpoint of T cell differentiation, after initial priming by DCs in the MLN.

Despite the importance of intestinal tissue macrophages for development of intestinal T cell populations, we are only now starting to understand the contribution of macrophage MHCII in these processes. A recent report showed that genetic ablation of MHCII on intestinal macrophages led to reduced T cell responses in *Salmonella* infection and highlighted a potential role in the regulation of oral tolerance^13^. In contrast, depletion of MHCII on other macrophage populations, for instance the microglia, had negligible effects on T cell responses^14^. Thus, there is a need to further clarify and characterise the mechanisms by which MHCII expression on intestinal macrophages is induced and maintained.

One factor that may contribute to the high levels of MHCII on intestinal macrophages may be their ontogeny. The intestine represents a somewhat unique case in that a major portion of the macrophage population requires constant replenishment by blood monocytes, which have been reported to upregulate MHCII upon entry^15,16^. However, the intestinal LP also contains a population of long-lived prenatally-derived macrophages^17^. Although phenotypically similar, the two macrophage populations can be differentiated by expression of the phosphatidylserine receptor T-cell immunoglobulin and mucin domain containing (Tim)4, which has been utilised as a marker for the prenatal origin of macrophages, both in the intestine and other tissues^17–19^.

Alternatively, the high level of MHCII expression on intestinal macrophages may reflect their presence at the mucosal surface. Macrophages residing within barrier tissues encounter very different stimuli from those in internal organs, which could affect MHCII expression independently of ontogeny. Indeed, both the microbiota and the intestinal epithelium can impact the phenotype and function of gut macrophages^13,20^. Importantly, even within the intestinal mucosa, there are differences between the microenvironments and immunomodulatory stimuli of the small intestine (SI) and the colon. The colon is dominated by microbial antigens and metabolites, whereas the SI contains comparatively few bacteria but is exposed to food-derived antigens and dietary metabolites^21,22^. Nevertheless, microbial colonisation influences the formation and function of the immune system throughout the intestinal tract.

In this study, we address the mechanisms controlling MHCII expression on intestinal macrophages, as well as its impact on local T cell populations. We demonstrate that the expression of MHCII on intestinal macrophages is controlled by multiple factors including macrophage ontogeny, tissue of residence and environmental stimuli. Macrophages in the SI, independently of ontogeny, maintained high expression of MHCII from early life into adulthood. In contrast, colonic macrophages displayed differential regulation of MHCII expression based on ontogeny and presence of microbial stimuli. Moreover, we demonstrate that MHCII expression on SI macrophages promotes the maintenance of tissue CD4^+^ T cell populations, suggesting that cognate macrophage-CD4^+^ T cell interactions may tune intestinal T cell homeostasis.

## Results

### Expression of MHCII on intestinal macrophages is dependent on ontogeny and tissue of residence

**Figure 1:**
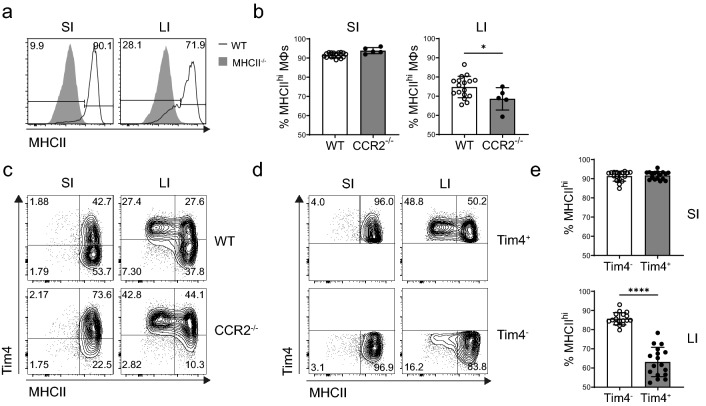
MHCII expression on different subsets of intestinal macrophages. (**a**) Representative histograms showing MHCII expression (black line) on macrophages from small (SI) or large (LI) intestine, gated as live CD45^+^ CD11b^+^ SiglecF^-^ Ly6G^-^ Ly6C^-^ CD64^+^ cells. The shaded histograms represent control staining of MHCII^-/-^ macrophages from the indicated tissue. (**b**) Proportion of MHCII^hi^ macrophages (gated as in a) in the SI or LI of wild-type (WT, N = 17) or CCR2^-/-^ mice (N = 5). (**c**) Representative FACS plots of Tim4 and MHCII expression on SI and LI macrophages in WT and CCR2^-/-^ mice. (**d**) Example gating on Tim4^+^ and Tim4^-^ macrophage subpopulations in the SI and LI, used for quantification of MHCII^hi^ macrophages within the two subsets. (**e**) Proportion of MHCII^hi^ macrophages within the Tim4^-^ and Tim4^+^ subsets of SI and LI macrophages (N = 17). In (**b**) and (**e**) the graphs represent the mean ± SD of data pooled from three (for WT mice) or two (for CCR2^-/-^ mice) independent experiments, with each dot representing an individual mouse. Groups were compared using a Mann–Whitney U test, with asterisks denoting statistical significance (*p ≤ 0.05, ****p ≤ 0.0001).

While many populations of tissue macrophages express MHCII, intestinal macrophages generally exhibit highest levels of surface MHCII (Supplementary Fig. 1). However, the expression of MHCII differs in the small intestine (SI) and colon. In the SI lamina propria (LP), nearly all macrophages are MHCII^hi^, while the colonic LP contains both a MHCII^hi^ population and a notable population of MHCII^lo^ macrophages (Fig. 1a). Recent studies have shown that intestinal macrophages represent an ontogenically heterogeneous population which comprise cells continually replenished by blood monocytes as well as long-lived, prenatally-derived macrophages^16,17^. To test whether MHCII expression varies with macrophage ontogeny, we analysed CCR2^-/-^ mice, which have a severely impaired monocyte egress from the bone marrow. Indeed, the proportion of MHCII^hi^ macrophages was further decreased in the colons of CCR2^-/-^ mice, indicating their monocyte origin (Fig. 1b,c). To further validate this, we used Tim4 as a surrogate marker of prenatal macrophage ontogeny^17^ and assessed MHCII expression on different gut macrophage tissue populations in wild-type (WT) mice (Fig. 1d). In the SI, nearly all macrophages expressed high levels of MHCII regardless of their ontogeny. In contrast, colonic macrophages showed differential MHCII expression based on ontogeny. While virtually all Tim4^-^ monocyte-derived macrophages were MHCII^hi^, a notably smaller proportion of Tim4^+^ prenatally-derived cells were MHCII^hi^ in the colonic LP (Fig. 1e). Therefore, in the colon, but not the SI, macrophage MHCII expression is partially dependent on their origin, with prenatally-derived Tim4^+^ cells containing a population of MHCII^lo^ macrophages.

### Intestinal macrophages acquire MHCII with different kinetics in early life

**Figure 2:**
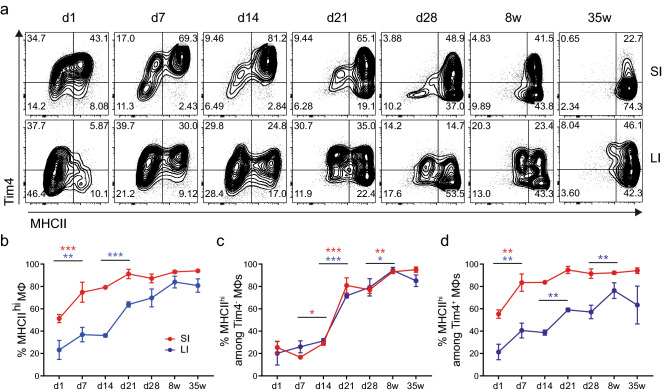
Differential kinetics of MHCII expression on macrophages in the small intestine and colon during neonatal development. (**a**) Representative FACS plots of MHCII and Tim4 expression on SI and LI macrophages of wild-type SPF mice at the indicated times after birth. (**b-d**) The graphs show the percentage of MHCII^hi^ cells within the total macrophages (**b**), or among Tim4^-^ (**c**) or Tim4^+^ (**d**) populations in the SI (red) and LI (blue) at indicated time-points after birth. Each point represents the mean ± SD of four mice from two independent experiments. Statistical comparisons were performed using two-way ANOVA with Bonferroni’s multiple comparison test with a Sidak correction. Asterisks denote statistical significance between adjacent time points (*p ≤ 0.05, **p ≤ 0.01, ***p ≤ 0.001).

To further understand how the establishment of intestinal macrophage populations affected MHCII expression, we next analysed the expression of MHCII on gut macrophages in early life. The expression of MHCII was monitored at 1, 7 and 14 days after birth, immediately after weaning (at day 21) and 28 days after birth (Fig. 2a). These were compared with data from adult mice at 8 and 35 weeks of age. Even directly after birth, ~ 50% of SI and ~ 20% of colonic macrophages expressed high levels of surface MHCII. In both SI and colon, the proportion of MHCII^hi^ macrophages further increased between days 1 and 7. In the SI, this resulted in a population of macrophages that was already ~ 80% MHCII^hi^ and steadily increased into adulthood, with a notable but non-significant increase at weaning. In the colon, the increase at day 7 was followed by another significant rise at weaning, which resulted in high levels of MHCII expression on ~ 60% of colonic macrophages. Subsequently, the proportion of MHCII^hi^ colonic macrophages underwent only a minor and gradual increase into adulthood (Fig. 2b).

The dynamics of MHCII expression on macrophages were markedly different according to their ontogeny. In both tissues, there was a significant increase in the proportion of MHCII^hi^ cells in the Tim4^-^ compartment at weaning (Fig. 2c). This coincides with the previously reported substantial increase in recruitment of monocytes into the gut mucosa which lose Ly6C expression and upregulate surface MHCII^15,16,23^. Notably, the kinetics of accumulation of MHCII^hi^ cells among monocyte-derived macrophages were very similar in the SI and colon.

In contrast, the increase in MHCII^hi^ cells among prenatally-derived Tim4^+^ macrophages followed different kinetics in the two organs. The proportion of MHCII^hi^ cells among the Tim4^+^ macrophages was already higher in the SI compared to the colon at day 1 after birth. The rise in the proportion of MHCII^hi^ Tim4^+^ macrophages mirrored that of the overall macrophage population. There was a significant increase immediately after birth in both SI and colon, a further increase at weaning and a significant increase between 28 days and adulthood in colonic macrophages (Fig. 2d). These observations suggested that prenatally-derived macrophages may respond to environmental changes by upregulating MHCII expression.

In summary, our data indicate that the expression of MHCII on the overall intestinal macrophage population is controlled by two distinct processes. Firstly, by the influx of monocytes, which has its peak at weaning and leads to the development of a uniformly MHCII^hi^ Tim4^-^ population in both the SI and the colon. Secondly, by an increase of MHCII expression amongst prenatally-derived Tim4^+^ macrophages.

### MHCII expression on macrophages in the colon, but not the small intestine, is controlled by the microbiota

**Figure 3:**
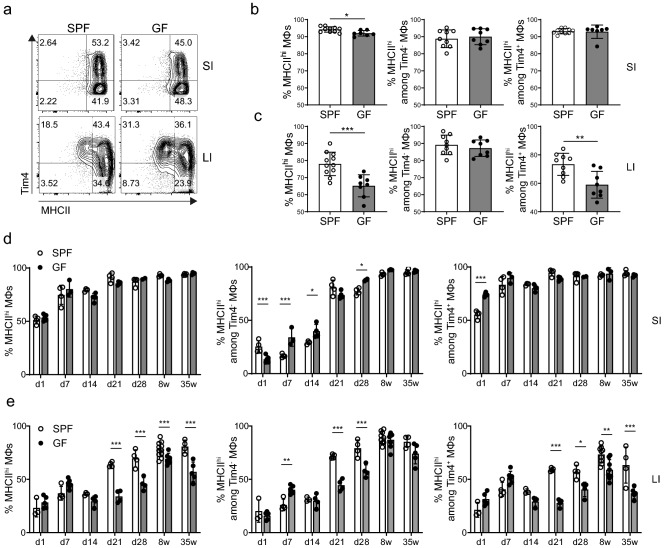
MHCII expression on colonic macrophages is reduced in germ-free mice. (**a**) Representative FACS plots of MHCII and Tim4 expression on SI and LI macrophages of wild-type SPF or germ-free (GF) mice. (**b, c**) Plots indicate the frequency of MHCII^hi^ cells within the total macrophages, or among Tim4^-^ or Tim4^+^ populations in the SI (**b**) and LI (**c**) of SPF (white bars) or GF (filled bars) eight weeks after birth. The graphs represent the mean ± SD of data pooled from three independent experiments, with each dot representing an individual mouse. Statistical comparisons were performed using the Mann–Whitney U test. (**d, e**) Plots indicate the frequency of MHCII^hi^ cells within the total macrophages, or among Tim4^-^ or Tim4^+^ populations in the SI (**d**) and LI (**e**) at the indicated time-points after birth. The graphs represent the mean ± SD of data pooled from three experiments performed at separate occasions, with each dot representing an individual mouse (N = 4, except GF d28 where N = 2). Statistical comparisons were performed using two-way ANOVA with Bonferroni’s multiple comparison test with a Sidak correction. Note that data from (**b, c**) are included as the 8 week time points in (**d, e**) for comparative purposes. For all panels, asterisks denote statistical significance (*p ≤ 0.05, **p ≤ 0.01, ***p ≤ 0.001).

The most notable changes in colonic macrophage MHCII expression correlated with the periods of major alterations in the intestinal microbiota: immediately after birth and at weaning. Therefore, we hypothesised that the intestinal microbiota had a direct effect on MHCII expression by intestinal macrophages. To directly assess whether the microbiota affects MHCII expression we analysed intestinal macrophages of germ-free (GF) adult animals. While SI macrophages were virtually all MHCII^hi^, we observed a small decrease in the frequencies of total MHCII^hi^ macrophages in the SI of GF mice which, while statistically significant, involved minimal differences in MHCII expression level, and could not be attributed to either Tim4^-^ or Tim4^+^ populations (Fig. 3a,b).

However, in line with previously published data^16^, we observed a significant decrease in the frequency of colonic MHCII^hi^ macrophages in GF mice when compared to control mice housed under specific pathogen free (SPF) conditions (Fig. 3a,c). Strikingly, this difference was entirely due to a reduced percentage of MHCII^hi^ cells among Tim4^+^, but not Tim4^-^ macrophages. This suggests that, while monocyte-derived macrophages express high levels of surface MHCII regardless of the status of the gut microbiota, MHCII expression on colonic prenatally-derived macrophages is dependent on microbiota-derived signals.

We next went on to characterise the kinetics of MHCII expression in early life in the absence of microbiota. Overall, SI macrophages in GF mice did not show any overt differences in kinetics of MHCII expression when compared to those of SPF mice, although minor differences could be detected within the Tim4^-^ macrophages pre-weaning, and in Tim4^+^ macrophages directly after birth (Fig. 3d). In contrast, colonic macrophages exhibited a significant reduction in the proportion of MHCII^hi^ cells, which was most pronounced at weaning, continued into adulthood and could also be detected in 35-week-old mice (Fig. 3e). Surprisingly, the difference in the proportion of MHCII^hi^ macrophages in the colons of SPF and GF mice at weaning could be observed in both Tim4^-^ and Tim4^+^ compartments. It had previously been suggested that germ-free mice show a delayed kinetics of monocyte infiltration into the colon ^16^, which may account for the observed decrease in MHCII^hi^ Tim4^-^ cells. However, while monocyte-derived Tim4^-^ macrophages eventually reached the levels of MHCII expression observed in adulthood, the proportion of MHCII^hi^ cells in the Tim4^+^ population remained significantly reduced in GF mice.

### Disruption of the microbiota results in downregulation of MHCII expression on colonic Tim4^+^ macrophages

**Figure 4:**
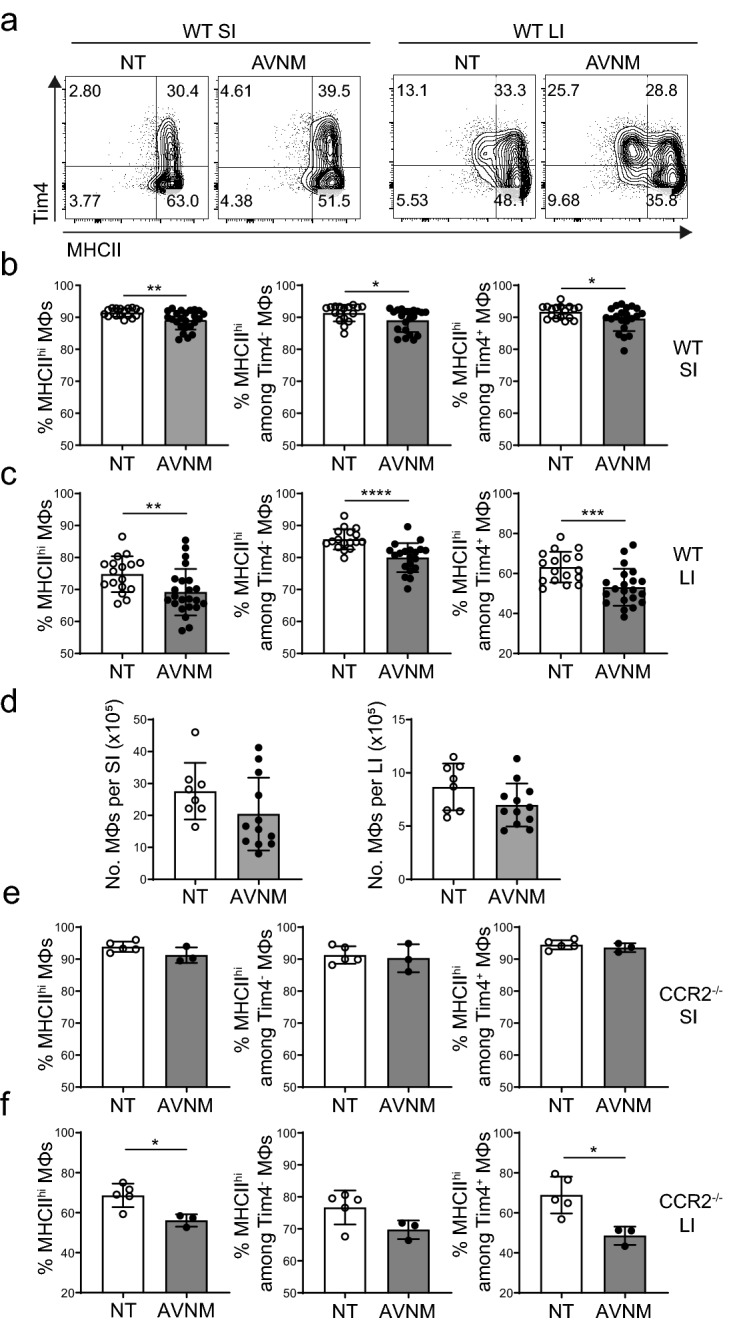
Antibiotics treatment reduces the proportion of colonic MHCII^hi^ macrophages. Mice were treated for seven days with a cocktail of antibiotics containing Ampicillin, Vancomycin, Neomycin and Metronidazole (AVNM) or non-treated as a control (NT). (**a**) Representative FACS plots of MHCII and Tim4 expression on SI and LI macrophages of antibiotic-treated (AVNM) or untreated (NT) wild-type mice. Plots indicate the frequency of MHCII^hi^ cells within the total macrophages, or among Tim4^-^ or Tim4^+^ populations in the SI (**b**) and LI (**c**) of WT mice. (**d**) Absolute numbers of macrophages in the SI and LI of antibiotic-treated (AVNM) or untreated (NT) wild-type mice. (**e, f**) Plots indicate the frequency of MHCII^hi^ cells within the total macrophages, or among Tim4^-^ or Tim4^+^ populations in the SI or LI of or CCR2^-/-^ mice. Results are presented as the mean ± SD of data from six (**b, c;** N(NT) = 17, N(AVNM) = 24), three (**d**; N(NT) = 8, N(AVNM) = 12) or two (**e, f;** N(NT) = 5, N(AVNM) = 3) independent experiments, with each dot representing an individual mouse. Statistical comparisons were performed using the Mann–Whitney U test. For all panels, asterisks denote statistical significance (*p ≤ 0.05, **p ≤ 0.01, ***p ≤ 0.001).

Since we established that colonic prenatally-derived macrophages exhibit a defect in upregulation of MHCII in the absence of intestinal microbiota, we next went on to assess whether the maintenance of macrophage MHCII expression relies on continual microbial signals. To disrupt the steady state microbiota, mice were treated ad libitum for seven days with a cocktail of broad-spectrum antibiotics. We observed minimal effects on SI macrophages (Fig. 4a,b) but a significant dysregulation of MHCII expression on colonic macrophages (Fig. 4a,c). Both Tim4^-^ and Tim4^+^ colonic macrophage subsets showed a significant reduction in the proportion of MHCII^hi^ cells. However, as seen in GF mice, the effect was of greatest magnitude on Tim4^+^ macrophages (Fig. 4c). Since the number of colonic macrophages was unaffected by antibiotics treatment (Fig. 4d), it is likely that the drop in the percentage of MHCII^hi^ macrophages was due to downregulation of surface MHCII expression.

To exclude the possibility that the reduction in MHCII^hi^ cells was due to replacement with monocyte-derived macrophages expressing low levels of MHCII, we repeated the experiment with CCR2^-/-^ mice, which have minimal numbers of monocyte-derived macrophages. We observed no changes in the SI but a significant decrease in the proportion of MHCII^hi^ Tim4^+^ colonic macrophages in CCR2^-/-^ mice after antibiotics treatment, confirming that the effect observed in WT mice was largely independent of monocyte recruitment (Fig. 4e,f). Therefore, the gut microbiota affects not only MHCII expression, but also MHCII maintenance on a subset of Tim4^+^ macrophages in the colon.

### Microbiota maintains MHCII expression on colonic macrophages through MyD88 signalling

Recent publications have emphasised the impact of bacterial metabolites on macrophage phenotype in the intestine, but also in the liver. In particular, short-chain fatty acids (SCFAs) have been shown to play a vital role in the maintenance of macrophage phenotype in the steady state gut^20^. To directly test whether the antibiotic-induced decrease in MHCII^hi^ Tim4^+^ macrophages was due to a lack of SCFAs, we supplemented the drinking water with a cocktail of butyrate, acetate and propionate throughout the seven day antibiotic treatment. However, we did not observe a difference in the proportion of SI or colonic MHCII^hi^ macrophages in the SCFA-supplemented group compared to mice that received antibiotics alone (Supplementary Fig. 2).

**Figure 5:**
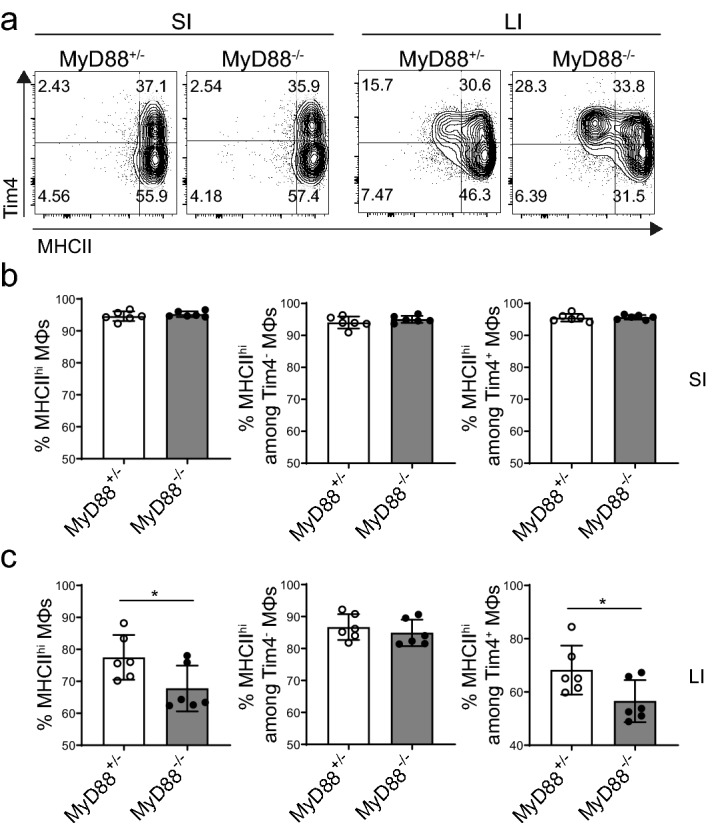
Decreased proportion of colonic MHCII^hi^ macrophages in MyD88 deficient mice. (**a**) Representative FACS plots of MHCII and Tim4 expression on SI and LI macrophages of MyD88^-/-^ or littermate control MyD88^+/-^ mice. Plots indicate the frequency of MHCII^hi^ cells within the total macrophages, or among Tim4^-^ or Tim4^+^ populations in the SI (**b**) and LI (**c**) of MyD88^+/-^ control mice (white bars) or MyD88^-/-^ mice (filled bars). Results are presented as the mean ± SD of data pooled from two independent experiments, with each dot representing an individual mouse (N = 6). Statistical comparisons were performed using a Mann–Whitney U test. Asterisks denote statistical significance (*p ≤ 0.05).

We next hypothesised that the intestinal microbiota may contribute to the maintenance of MHCII expression on Tim4^+^ colonic macrophages by direct stimulation through pattern recognition receptors, such as toll-like receptors (TLRs). To test this hypothesis, we analysed macrophages from the intestines of MyD88^-/-^ mice, which have severely impaired TLR signalling. Strikingly, we observed a phenotype similar to that of GF and antibiotic-treated mice, with a reduction in the proportion of MHCII^hi^ on colonic but not SI macrophages compared to littermate MyD88^+/-^ controls (Fig. 5a–c). Moreover, the decreased MHCII expression was almost entirely attributable to the Tim4^+^ macrophage subset. Collectively, our data are consistent with the hypothesis that the colonic microbiota contributes to maintaining high levels of surface MHCII expression on colonic macrophages through MyD88-mediated signalling.

### Macrophage expression of MHCII supports intestinal CD4^+^ T cell maintenance

Finally, we went on to examine the role of MHCII expression on intestinal macrophages on the gut T cell population. Technically, addressing the role of antigen presentation by macrophages is difficult, as the majority of macrophage-specific genetic models also affect DCs^24^. We therefore devised a mixed bone marrow chimaera system where we took advantage of impaired monocyte recruitment of CCR2^-/-^ progenitors. We exposed CD45.1/CD45.2 mice to a lethal dose of radiation and reconstituted them with a 90:10 mix of CCR2^-/-^ and MHCII^-/-^ bone marrow cells. Since CCR2 is required for monocyte egress from the bone marrow, replenishment of intestinal macrophages after irradiation predominantly occurs from the MHCII^-/-^ progenitors, while replenishment of monocyte-independent populations, including DCs, would be largely unaffected. Indeed, the intestines of these mice contained predominantly MHCII^-^ macrophages, while the DC population was largely MHCII^+^ (Supplementary Fig. 3a–c). As controls, we used mice reconstituted with either a 90:10 mix of CCR2^-/-^ and wild-type bone marrow cells, where all cells can express MHCII, or with a 90:10 mix of WT and MHCII^-/-^ bone marrow cells, where 90% of macrophages express MHCII.

**Figure 6:**
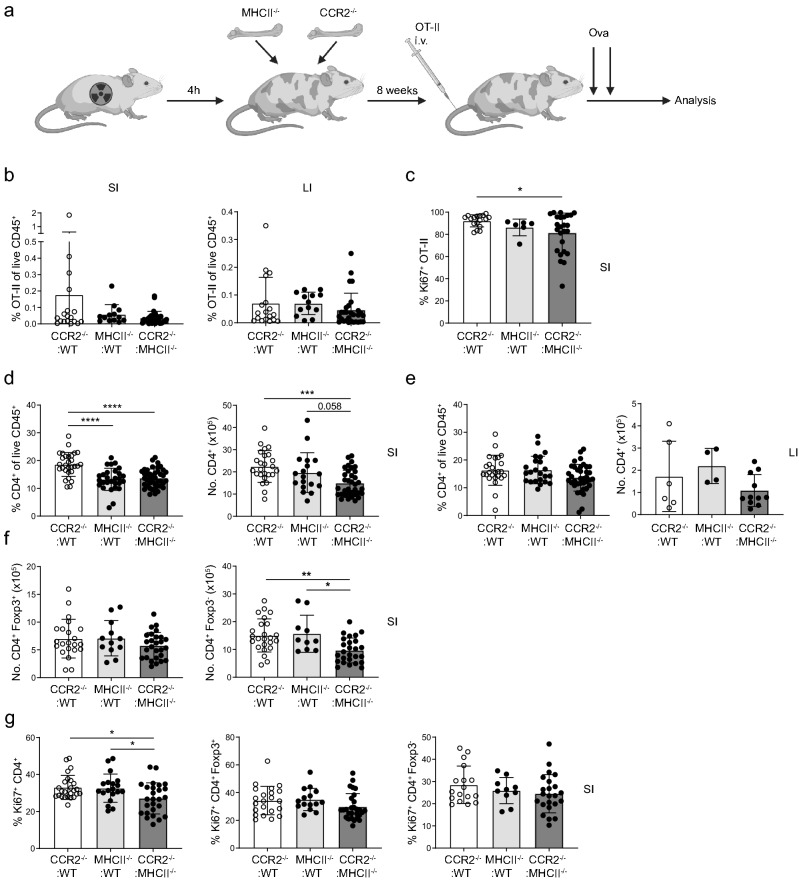
Reduction of the CD4^+^ T cell population in the small intestines of mice with a macrophage-specific MHCII deficiency. (**a**) Schematic representation of the experimental design. Indicated bone marrow chimaeric mice were given Dendra-2 expressing OT-II cells by adoptive transfer followed by two doses of oral ovalbumin. Five days later OT-II and endogenous T cells were analysed by flow cytometry. (**b**) Proportion of OT-II T cells, gated as live, CD4^+^ Dendra-2^+^ TCRVα2^+^Vβ5^+^ cells, among live leukocytes of the SI and LI of the indicated chimaeric mice. (**c**) Proportion of Ki67^+^ OT-II T cells in the SI of the indicated chimaeric mice. Frequency and number of endogenous recipient CD45.1^+^ CD45.2^+^ CD4^+^ T cells in the SI (**d**) or LI (**e**) of the indicated chimaeric mice. (**f**) Absolute numbers of Foxp3^+^ and Foxp3^-^ endogenous recipient CD45.1^+^ CD45.2^+^ CD4^+^ T cells in the SI of the indicated chimaeric mice. (**g**) Proportion of Ki67^+^ cells amongst total or Foxp3^+^ and Foxp3^-^ fractions of endogenous CD45.1^+^ CD45.2^+^ CD4^+^ T cells in the SI of the indicated chimaeric mice. For all panels results are presented as the mean ± SD of data pooled from three independent experiments with each dot representing an individual mouse (CCR2^-/-^:WT N = 28, MHCII^-/-^:WT, N = 28, CCR2^-/-^:MHCII^-/-^, N = 40). Statistical comparisons were performed using one-way ANOVA with Tukey’s multiple comparison test. Asterisks denote statistical significance (*p ≤ 0.05, **p ≤ 0.01, ***p ≤ 0.001, ****p ≤ 0.0001).

The experimental and control bone marrow chimaeras were adoptively transferred with 5 × 10^6^ ovalbumin-specific OT-II CD4^+^ cells. A day later, the mice were given two doses of ovalbumin by oral gavage. Five days later, the SI, colon and MLNs were assessed for OT-II T cell frequencies (Fig. 6a). We observed no differences in the overall frequency of OT-II T cells in the SI, colon (Fig. 6b) or the MLN (data not shown) of mice with the macrophage-specific MHCII deficiency compared to control bone marrow chimaeras. This confirms that macrophage MHCII is not required for T cell priming or migration into the gut, as expected. However, we observed a small but significant decrease in the proportion of Ki-67^+^ OT-II T cells in the SI, indicating that local proliferation of newly arrived T cells might be impaired in the absence of MHCII on intestinal macrophages (Fig. 6c). However, due to the difficulty of assessing Ki67 on small numbers of OT-II T cells, we cannot exclude the possibility that this decrease is also present in the intestine of control MHCII^-/-^: WT chimaeras.

Interestingly, while there was no obvious difference in the expansion of antigen-specific T cells in the intestinal LP, we observed a significant decrease in the proportion and number of endogenous recipient T cells in the SI but not colonic LP of CCR2^-/-^: MHCII^-/-^ bone marrow chimaeras (Fig. 6d,e, Supplementary Fig. 3d). Moreover, the decrease in the number of SI CD4^+^ T cells appeared to be largely confined to the Foxp3^-^ population of effector/memory T cells (Fig. 6f). In contrast, the overall Treg numbers remained unaltered. The proliferation capacity of endogenous CD4^+^ T cells, as measured by Ki-67 staining, was significantly reduced in the SI of mice lacking macrophage MHCII expression, likely as a combined effect on both Foxp3^+^ or Foxp3^-^ compartments (Fig. 6g). Collectively, these data suggest that while MHCII on intestinal macrophages has little effect on development of new CD4^+^ T cell responses, it may support the long-term survival and/or homeostatic maintenance of LP-resident effector/memory CD4^+^ T cells.

**Figure 7:**
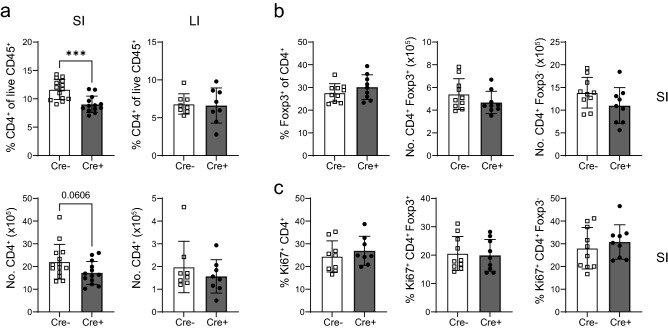
Reduction of the CD4^+^ T cell population following ablation of macrophage MHCII expression in the small intestine. (**a**) Proportion and absolute number of CD4^+^ T cells in the SI or LI of tamoxifen treated CX3CR1^CreER^ MHCII^fl/fl^ (Cre +) or MHCII^fl/fl^ (Cre-) littermate control mice. (**b**) Proportion of Foxp3^+^ and absolute number of Foxp3^+^ or Foxp3^-^ CD4^+^ T cells in the SI of tamoxifen treated CX3CR1^CreER^ MHCII^fl/fl^ (Cre +) or MHCII^fl/fl^ (Cre-) littermate control mice. (**c**) Proportion of Ki67^+^ cells amongst total or Foxp3^+^ and Foxp3^-^ fractions of CD4^+^ T cells in the SI of tamoxifen treated CX3CR1^CreER^ MHCII^fl/fl^ (Cre +) or MHCII^fl/fl^ (Cre-) control mice. For all panels results are presented as the mean ± SD of data pooled from three independent experiments, with each dot representing an individual mouse (N(Cre-) = 10, N(Cre +) = 9). Statistical comparisons were performed using the Mann–Whitney U test. Asterisks denote statistical significance (***p ≤ 0.001).

The irradiation necessary for bone marrow chimaera establishment in this system may have confounding effects on intestinal T cell populations, potentially affecting their overall survival. We therefore sought to establish a system of a macrophage-specific MHCII deficiency that does not rely on lethal radiation. To this end, we made use of the inducible CX3CR1^Cre-ER^ mice^25^, crossed onto the MHCII^fl/fl^ background. In these mice, application of tamoxifen results in the deletion of MHCII in all CX3CR1^+^ cells, which, in the intestine, is largely specific to the macrophage compartment (Supplementary Fig. 4a-c and^13^). Similarly to the results obtained from the bone marrow chimaera approach, we again observed a significant decrease in the overall frequency and a substantial decrease in the number of CD4^+^ T cells in the SI LP but not colonic LP of tamoxifen treated mice (Fig. 7a). In this system, there was no difference in the relative proportion or number of SI Foxp3^+^ and Foxp3^-^ cells (Fig. 7b). Moreover, the Ki-67 staining showed no difference between the SI of tamoxifen treated and control mice, indicating normal proliferation and suggesting a defect in homeostatic maintenance and survival (Fig. 7c).

Collectively, our data demonstrate that expression of MHCII on intestinal macrophages aids the homeostatic maintenance and survival of CD4^+^ T cells in the SI and may support the longevity of tissue-resident T cell populations.

## Discussion

A consensus has emerged over the last decade that tissue imprinting plays a major part in establishing the phenotype of the resident macrophage populations^26,27^. In parallel, a reframing of macrophage ontogeny revealed that macrophages in most tissues originate from a mix of prenatally-derived, long-lived cells as well as monocyte-derived cells of classical haematopoietic origin^17,28^. Overall, it appears that tissue of residence, rather than ontogeny, is the deciding influence on the phenotype and function of tissue resident macrophages. Indeed, in various tissues, local microenvironmental cues control the development of particular tissue-specific phenotypes^27,29–31^. In contrast, it is still uncertain to what extent development from distinct precursors may impact the phenotype and function of mature tissue macrophages.

Our data demonstrate that, in the intestine, ontogeny of gut macrophages contributes to the regulation of MHCII expression. In adult mice, prenatally-derived colonic Tim4^+^ macrophages showed heterogeneous expression of MHCII, while Tim4^-^ macrophages were exclusively MHCII^hi^, in both the small and large intestine. During neonatal development, there is a burst of monocyte recruitment around weaning^16^. In line with this, we observed an increase in Tim4^-^ macrophages at weaning, which corresponded to a major increase in MHCII expression. Additionally, we observed lower frequencies of MHCII^+^ macrophages in the intestinal tissues of CCR2^-/-^ mice, which have a severe defect in monocyte egress from bone marrow. These results suggest that MHCII expression is an obligate part of monocyte differentiation pathway into gut tissue macrophages. This corresponds to previous work that has demonstrated that monocytes infiltrating both the steady-state and inflamed intestine acquire MHCII expression as early as one day after entry^15,16,23^. In addition, in an experimental system of depletion of alveolar macrophages, monocyte-derived macrophages were able to replenish the niche and differentiate into cells with an almost identical transcriptional signature. Notably, among the few genes that were differentially expressed were MHCII-related genes, which were upregulated in monocyte-derived compared to *bona fide* alveolar macrophages^27^. These results further support the hypothesis that monocyte-derived macrophages have a propensity to express surface MHCII upon entry into peripheral tissues, although the molecular mechanisms controlling this process remain elusive.

In contrast to monocyte-derived cells, which invariably acquired MHCII, prenatally-derived, long-lived gut macrophages express little MHCII at birth and acquire it during neonatal development. Importantly, the upregulation of MHCII on prenatally-derived macrophages occurs with different kinetics and is controlled by distinct environmental stimuli in the SI and colon. In the SI, the weaning period resulted in ubiquitous MHCII expression by all macrophages, regardless of ontogeny, which was maintained into adulthood. In contrast, the percentage of colonic Tim4^+^ MHCII^hi^ macrophages was lower than in the SI throughout neonatal development. Even in adult animals, a substantial proportion of MHCII^lo^ macrophages could be observed, all of which were Tim4^+^, and more prevalent in CCR2^-/-^ animals, confirming their monocyte independent ontogeny. Therefore, the increase in MHCII^+^ macrophages reflects both an influx of monocyte-derived macrophages and an upregulation of MHCII expression on prenatally-derived cells. Notably, the increase in MHCII expression on colonic Tim4^+^ macrophages was affected by the microbial status of the mice. We observed decreased proportions of MHCII^hi^ macrophages in GF, antibiotic-treated and MyD88^-/-^ mice, mainly amongst Tim4^+^ prenatally-derived colonic macrophages. Therefore, distinct environmental cues regulate MHCII expression on gut prenatally-derived macrophages, with microbiota-derived signals being essential for MHCII maintenance in the colon but not the SI.

The microbiota produces a wide variety of metabolites which can profoundly affect the phenotype and function of host immune cells. In particular, SCFAs have been shown to exert a major influence on the phenotype and metabolism of tissue macrophages^20,32^. However, while we observed no difference in antibiotic-induced downregulation of MHCII upon co-administration of SCFAs, we cannot eliminate the possibility that SCFAs did not reach the colon in our experimental set-up and may play a role in maintaining colonic macrophage MHCII expression.

Apart from metabolites, the microbiota express and release a variety of other molecules which are directly sensed by pattern recognition receptors such as TLRs, expressed on immune cells. Indeed, the loss of TLR signalling in MyD88^-/-^ mice resulted in a significant decrease of Tim4^+^ MHCII^hi^ macrophages in the colon, but not the SI, recapitulating the phenotype observed in GF mice. We therefore hypothesise that microbial recognition through TLR signalling results in upregulation of MHCII expression on long-lived colonic macrophages. In line with this, several studies have reported TLR-dependent translocation of phagosomal MHCII to the cell surface^33,34^. Alternatively, the MyD88-dependent increase of MHCII may be driven by NFAT-induced CIITA activity which is enhanced upon TLR signalling^35,36^. It is worth noting however, that, in addition to TLRs, MyD88 is the key adaptor in IL-1, IL-18 and IL-33 signalling, which therefore may also play a role in regulating colonic macrophage MHCII expression. However, further in vivo experiments using specific agonists or knock-out models will be necessary to clarify the main MyD88-dependent pathways involved in this process.

It remains elusive which environmental stimuli affect the increase in MHCII expression on SI prenatally-derived macrophages observed after birth and at weaning. One possibility is that ingestion of food, initially milk, later solid food, may contain signals which regulate MHCII expression. Indeed, dietary metabolites such as retinoids and aryl-hydrocarbon receptor ligands have a wide range of immunomodulatory functions ^21,37^.

Nevertheless, it appears there is strong evolutionary pressure to maintain MHCII expression on SI macrophages from neonatal development into adulthood. We therefore used two different experimental systems of macrophage-specific MHCII deficiency to examine the effect on CD4^+^ T cells. While these approaches unfortunately do not let us distinguish between prenatal and monocyte-derived macrophage expression of MHCII, we primarily aimed to address the overall effect of macrophage MHCII deficiency. Indeed, we observed a decrease of CD4^+^ T cells in the SI but not the colon in the absence of macrophage-expressed MHCII, likely reflecting a defect in homeostatic proliferation, survival or maintenance.

The importance of intestinal macrophages as antigen presenting cells has long been a topic of interest and controversy. Many studies have tried to unravel the ability of macrophages to present antigen in a MHCII-dependent manner. Denning and colleagues showed that CD11b^+^CD11c^-^ SI cells were able to stimulate OT-II cell proliferation and differentiation into Foxp3^+^ Tregs in an antigen-dependent manner^38^. Similarly, colonic CX3CR1^+^ mononuclear cells stimulated IL-17A and IFN-γ production in OT-II cells *in vitro*^39^. In contrast, other reports found that intestinal macrophages had a poor capacity to induce proliferation of naïve OT-II cells^5^. One possible explanation for this discrepancy is that some macrophage preps may contain CD103^-^ CX3CR1^+^ cDC2s which, unlike macrophages, migrate in lymph and stimulate naïve T cells into a T helper (TH)1 and TH17 phenotype^2,4,5^. Indeed, macrophages are sedentary in intestinal LP, which is largely devoid of naïve T cells^40^. Therefore, any antigen presentation by gut macrophages is more likely to affect tissue-resident memory/effector populations. Indeed, using a similar model of macrophage-specific MHCII depletion a recent study demonstrated a role for macrophages in modulating CD4^+^ T cell responses in *Salmonella* infection^13^. Here we report a similar defect in intestinal CD4^+^ T cells in the absence of MHCII on macrophages, which may reflect cognate antigen presentation. However, we cannot exclude the possibility that macrophages may provide “tonic” MHCII signals which are thought to be necessary for T cell survival and maintenance^41^. In line with this, we did not observe an impairment in antigen-specific intestinal CD4^+^ OT-II T cells, following feeding of their cognate antigen in mice lacking macrophage MHCII expression. Interestingly, a similar study reported decreased numbers of OT-II Tregs in the SI of CX3CR1^CreER^ MHCII^fl/-^ mice. However, this was accompanied by a reduction in OT-II numbers in the MLN suggesting the possibility that OT-II priming may have been affected^13^. Further studies would be required to ascertain whether the T cells in the LP of mice with MHCII-deficient macrophages exhibit altered functionality, corresponding to reduced tonic signalling.

In this study we did not assess the impact of MHCII depletion on the function of gut macrophages themselves. The phenotype and function of intestinal macrophages can be modulated by T cell cytokine production or direct cell contact. Indeed, in a model of colitis, Tregs can restrain macrophage production of IL-23 and IL-1β in a LAG3-dependent manner, which may interact with MHCII on the surface of gut macrophages^42^. Thus, regulation of MHCII expression on intestinal macrophages may impact both the T cell response as well as macrophage function.

Collectively, our data suggest that expression of MHCII on intestinal macrophages depends on macrophage ontogeny, environmental stimuli and age. Furthermore, expression of MHCII on gut macrophages appears to have a role in maintaining a stable population of LP CD4^+^ T cells, particularly in the SI. Increased understanding of the processes controlling gut macrophage biology and their function in homeostasis of intestinal T cell populations may lead to the development of novel approaches to modulate their function such as therapies which may be used to enhance homeostatic T cell activity and promote gut health.

## Methods

### Mice

Wild-type C57BL/6, CX3CR1^+/gfp^, CCR2^-/-^, MyD88^-/-^ and OT-II mice crossed onto Vav-H2B-Dendra2 (VHD)^43^ background were bred and maintained under SPF conditions at the central animal facility of RWTH Aachen University. CX3CR1^CreER^ MHCII^fl/fl^ mice were generated by crossing CX3CR1^CreER^ mice^25^ (from Prof. Steffen Jung) with MHCII^fl/fl^ (from Prof. Charlotte Scott). Germ-free C57BL/6 mice were maintained under germ-free conditions at the central animal facility of RWTH Aachen University. All mice used were age-matched 8–10 weeks old unless indicated differently. Animal work was performed in compliance with ethics regulations of German Law for the Protection of Animal Welfare (Tierschutzgesetz), and the experimental protocols were specifically approved (84–02.04.2016.A285) by the North Rhine-Westphalia State Agency for Nature, Environment and Consumer Protection (LANUV). All animal experiments were performed in accordance with the recommendations in the ARRIVE guidelines.

### Cell isolation

Cells from the intestinal LP were isolated as described previously^4,44^. Briefly, the tissues were digested with 1 mg/ml Collagenase VIII (Sigma–Aldrich) (SI) or 0.85 mg/ml Collagenase V (Sigma-Aldrich), 1.25 mg/ml Collagenase D (Roche), 1 mg/ml Dispase (Gibco) and 30 μg/ml DNase I (Roche) (colon). Peritoneal cells were isolated by lavage with 4 ml PBS/3% FCS. Mice were perfused with PBS and liver, spleen, lungs and brain were removed and macerated. Lungs were digested with 20 μg/ml Liberase (Roche) and 30 μg/ml DNase (Roche). Spleens and brains were digested with 1 mg/ml Collagenase D (Roche) and 0.15 mg/ml DNAse. Liver was digested with 0.5 mg/ml Collagenase A (Roche) followed by Percoll (GE Healthcare) density centrifugation. The ventral and dorsal sheets of the ear were separated from the cartilage and incubated with 2.5 mg/ml Dispase (Gibco) to separate the epidermal and dermal sheets before incubation with 0.25 mg/ml Liberase (Roche) and 0.15 mg/ml DNase. Single cell suspensions were filtered through 100 μm strainers and resuspended in PBS/3% FCS for further analysis.

### Flow cytometry and cell sorting

For fluorescent staining, single cell suspensions were blocked with 5% rat serum for 5 min. Fluorochrome-conjugated monoclonal antibodies were used: CD103 (2E7), CD115 (AFS98), CD11b (M1/70), CD11c (N418), CD4 (RM4-5), CD45 (30-F11), CD45.1 (A20), CD45.2 (104), CD64 (X54-5/7.1), F4/80 (BM8), Ly6C (HK1.4), Ly6G (1A8), MHCII I-A/I-E (M5/114.15.2), TCRβ (H57-597), TCR Vα2 (B20.1), Tim4 (RMT4-54) (all from Biolegend), TCR Vβ5.1 (MR9-4, BD), SiglecF (E50-2440, BD). Cell viability was established with 7AAD (Biolegend) or Fixable Viability Dye eFluor™ 780 (eBioscience). For intracellular staining, the Foxp3/Transcription Factor Staining Buffer Set (eBioscience) was used according to manufacturer’s instructions. FoxP3 (FJK-16 s, eBioscience) and Ki67 (SolA15, eBioscience) were used for intracellular staining. Flow cytometry was performed using a LSRFortessa (BD) and analysed with FlowJo software (FlowJo). Cell sorting was performed using a FACSAriaII (BD).

### Antibiotics treatment

Eight-week-old mice were subjected to a cocktail of antibiotics in the drinking water, containing 1 g/l ampicillin (Sigma–Aldrich), 0.5 g/l vancomycin (HEXAL), 1 g/l neomycin (Sigma–Aldrich) and 1 g/l metronidazole (Braun). Mice received antibiotics ad libitum for seven days. In some experiments, the drinking water was supplemented with a mixture of 200 mM butyrate (Sigma–Aldrich), 200 mM propionate (Sigma–Aldrich) and 200 mM acetate (Carl-Roth).

### Tamoxifen administration

Tamoxifen (Sigma–Aldrich) was dissolved in corn oil (Sigma–Aldrich). Mice received four doses of 0.2 mg tamoxifen by oral gavage every second day.

### Bone marrow chimaeras

Eight-week-old C57BL/6 CD45.1/CD45.2 mice were exposed to 10 Gy radiation using a Faxitron CP-160. Mice were reconstituted by intravenous injection of 5 × 10^6^ cells from a 9:1 mixture of CCR2^-/-^CD45.1 and MHCII^-/-^CD45.2 BM. Control animals received either a 9:1 mix of WT CD45.1 and MHCII^-/-^ CD45.2 cells or CCR2^-/-^ CD45.1 and WT CD45.2 cells. Irradiated mice received 1.25 mg/ml of the cotrimoxazole (Ratiopharm) in drinking water for two weeks. Mice were left to recover at least 8 weeks before the start of the experiments.

### Adoptive transfer

LNs of OT-II VHD mice were isolated, mashed and filtered through a 40 μm cell strainer (Falcon). Naïve CD4^+^ T cells were sorted and 5 × 10^6^ cells were transferred intravenously into recipient mice. One day later, mice received 50 mg ovalbumin (Grade III, Sigma–Aldrich) by oral gavage for two consecutive days.

### Data presentation and statistical analysis

Statistical analysis was performed and the graphs plotted using GraphPad Prism. Schematic in Fig. 6a was created with BioRender.com. Gaussian distribution was tested using Kolmogorov–Smirnov test. All data are presented as mean ± SD and P values of less than 0.05 were considered significant. Statistical tests used and levels of significance are detailed in each figure legend.

## Supplementary Information

Supplementary Information.

## Data Availability

The datasets generated during and/or analysed during the current study are available from the corresponding author on reasonable request.
