# Supplementary material for: MHCII expression on gut macrophages supports T cell homeostasis and is regulated by microbiota and ontogeny
Source: Sci Rep. 2023 Jan 27;13:1509. doi: 10.1038/s41598-023-28554-8 (PMC9883227; doi:10.1038/s41598-023-28554-8)
Supplement: Supplementary file 1 — Supplementary Information. [file 41598_2023_28554_MOESM1_ESM.pdf]

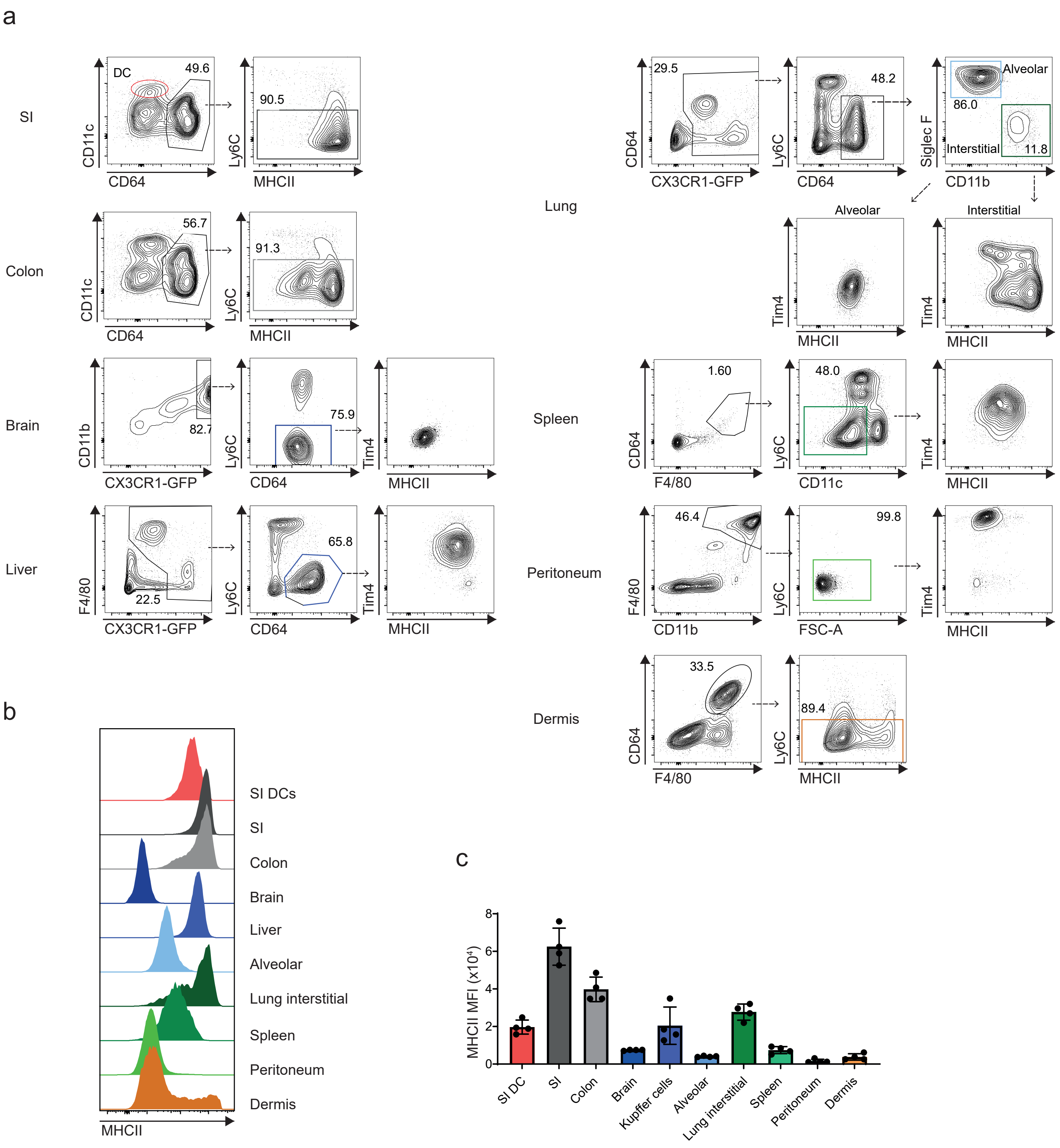

Supplementrary Figure 1

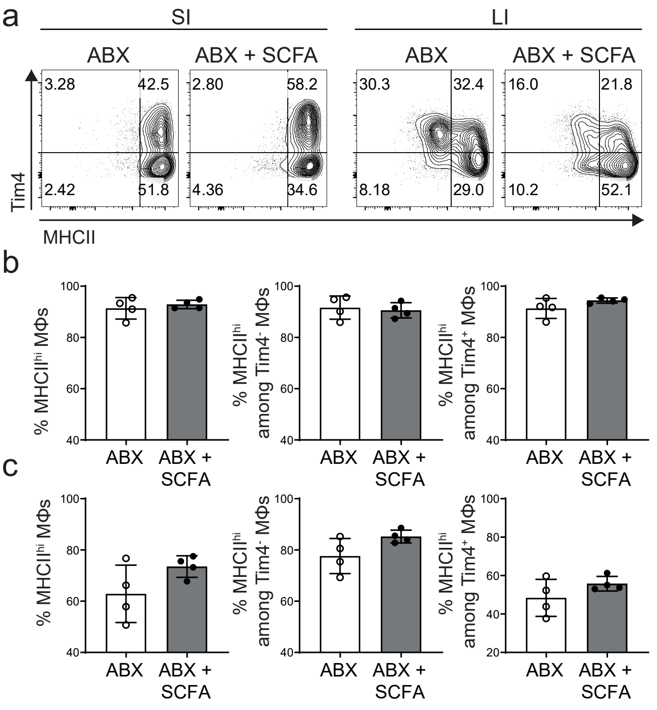

Supplementrary Figure 2

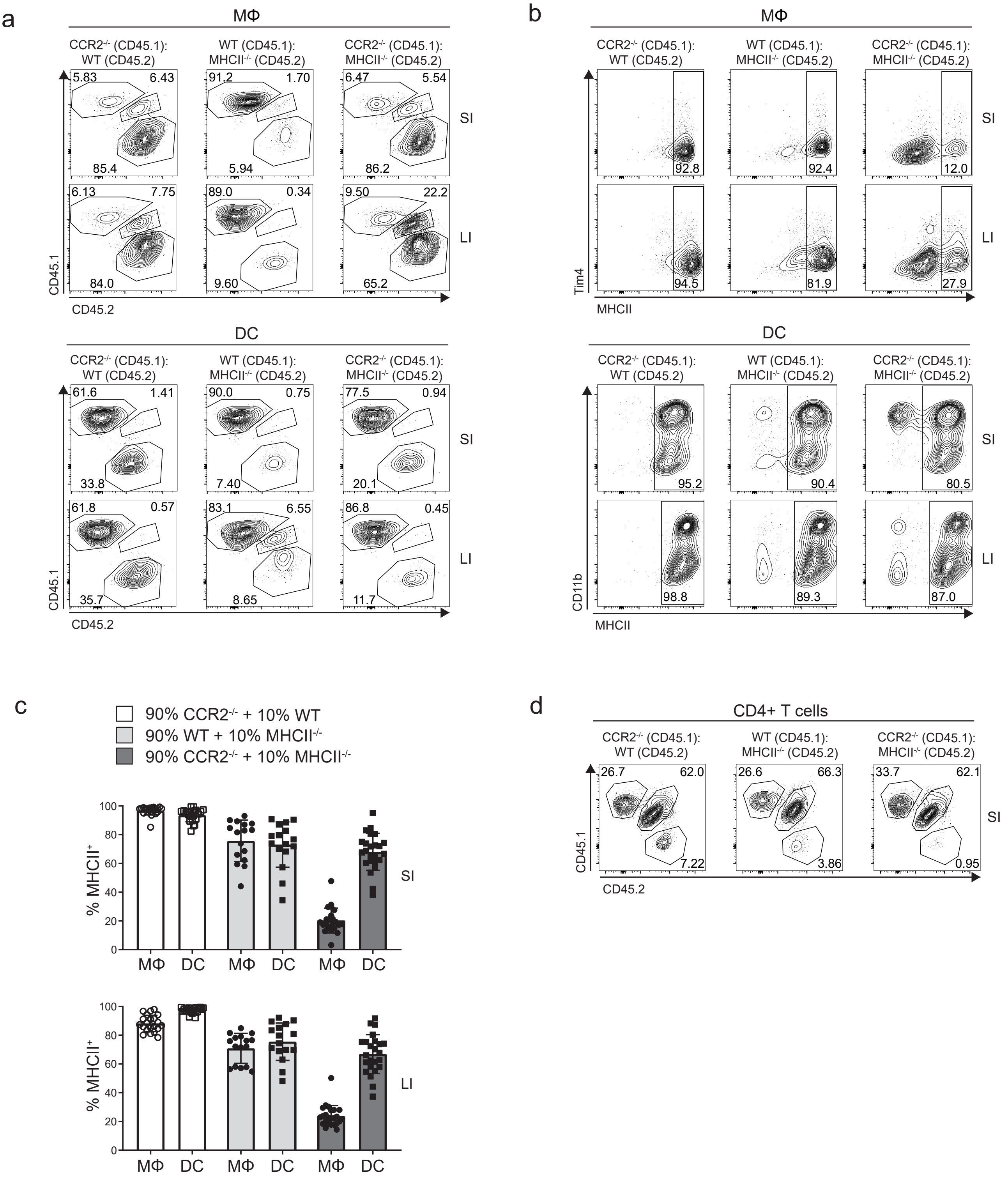

Supplementrary Figure 3

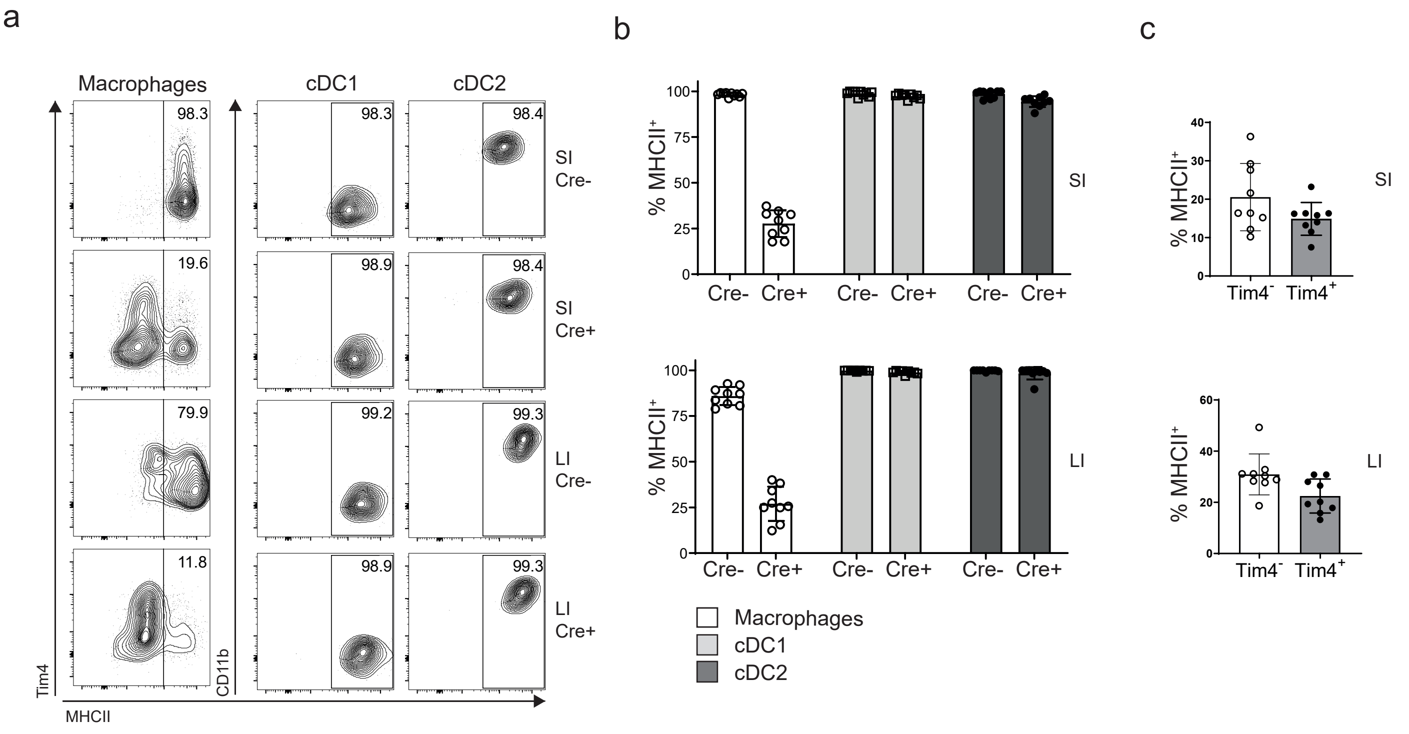

Supplementrary Figure 4

Supplementary Figure 1 MHCII expression on tissue macrophages. (**a**) Gating of macrophage populations from the indicated tissues of CX3CR1$^{+/gfp}$ mice. All populations were initially pre-gated on live single CD45^{+} cells and then gated on the specified markers. (**b**) Offset overlay histograms of surface MHCII expression on the indicated macrophage populations. The red histogram shows the expression on SI DCs for comparative purposes. (**c**) Mean fluorescence intensity (MFI) of MHCII on the indicated tissue macrophages. Results are presented as the mean ± SD of data from one experiment, with each dot representing an individual mouse (N=4).

Supplementary Figure 2 SFCAs do not alter antibiotics-induced MHCII downregulation on colonic macrophages. Mice were treated for seven days with a cocktail of antibiotics (as in Figure 4) with or without the addition of short chain fatty acids (SCFAs) acetate, propionate, and butyrate. (**a**) Representative FACS plots of MHCII and Tim4 expression on SI and LI macrophages of antibiotic-treated wild-type mice with or without the addition of SCFAs. (**b, c**) Plots indicate the frequency of MHCII^{hi} cells within the total macrophage, or among Tim4^{-} or Tim4^{+} populations in the SI (**b**) and LI (**c**). Results are presented as the mean ± SD of data pooled from two independent experiments, with each dot representing an individual mouse (N=4). Statistical comparisons were performed using a Mann-Whitney U test.

Supplementary Figure 3 Bone marrow chimaera model of macrophage-specific MHCII deficiency. (**a**) Reconstitution of macrophages (MΦ, upper panels), gated as live CD45^{+} CD11b^{+} Ly6C^{-} CD64^{+} cells and dendritic cells (DC, lower panels), gated as live CD45^{+} CD11c^{hi} Ly6c^{-} Ly6g^{-} SiglecF^{-} CD64^{-} cells, in the small intestines (SI) and large intestines (LI) of indicated bone marrow chimaeras. Representative plots (**b**) and summary graphs (**c**) of the proportion of MHCII^{+} macrophages (MΦ), and dendritic cells (DC), in the SI and LI of indicated bone marrow chimaeras. Results are presented as the mean ± SD with each dot representing an individual mouse (CCR2$^{-/-}$:WT N=21, MHCII$^{-/-}$:WT, N=16, CCR2$^{-/-}$:MHCII$^{-/-}$, N=24). (**d**)

Reconstitution efficiency of $CD4^+$ T cells, gated as $CD45^+$ $TCR\beta^+$ $CD4^+$ in the small intestines of the indicated bone marrow chimaeras. In (**a**) and (**d**), $CD45.1^+$ $CD45.2^+$ cells represent remaining endogenous recipient cells.

Supplementary Figure 4 Inducible deletion of MHCII on intestinal macrophages. Representative FACS plots (**a**) and summary graphs (**b**) showing the proportion of $MHCII^+$ macrophages (MΦ), gated as live $CD45^+CD11b^+$ $Ly6C^-CD64^+$ cells and dendritic cells (DC), gated as live $CD45^+$ $CD11c^{hi}$ $Ly6c^-$ $Ly6g^-$ $SiglecF^-$ $CD64^-$ cells and either as $CD103^+$ $CD11b^-$ (cDC1) or $CD103^+$ $CD11b^+$ (cDC2) in the small intestine and colon of $CX3CR1^{CreER}$ $MHCII^{fl/fl}$ mice (Cre+) or $MHCII^{fl/fl}$ (Cre-) controls, following tamoxifen treatment. (**c**) Percentage of $MHCII^+$ cells in the small intestines or colons of $CX3CR1^{CreER}$ $MHCII^{fl/fl}$ mice (Cre+) mice among either the $Tim4^-$ or $Tim4^+$ subset of macrophages, gated as in Figure 1d. Results are presented as the mean ± SD of data pooled from two independent experiments, with each dot representing an individual mouse (N=9).
